# Supplementary material for: Iterative improvement in the automatic modular design of robot swarms
Source: PeerJ Comput Sci. 2020 Dec 7;6:e322. doi: 10.7717/peerj-cs.322 (PMC7924708; doi:10.7717/peerj-cs.322)
Supplement: Supplemental Information 3 [file peerj-cs-06-322-s003.zip › argos3/doc/api/standalone/a00571_source.html]

ARGoS: plugins/simulator/entities/ground\_sensor\_equipped\_entity.h Source File


- Main Page
- Related Pages
- Namespaces
- Classes
- Files

- File List
- File Members

# plugins/simulator/entities/ground\_sensor\_equipped\_entity.h

Go to the documentation of this file.

```
00001 
00007 #ifndef GROUND_SENSOR_EQUIPPED_ENTITY_H
00008 #define GROUND_SENSOR_EQUIPPED_ENTITY_H
00009 
00010 namespace argos {
00011    class CGroundSensorEquippedEntity;
00012 }
00013 
00014 #include <argos3/core/utility/math/vector3.h>
00015 #include <argos3/core/simulator/entity/entity.h>
00016 #include <argos3/core/simulator/entity/embodied_entity.h>
00017 #include <map>
00018 
00019 namespace argos {
00020 
00021    class CGroundSensorEquippedEntity : public CEntity {
00022 
00023    public:
00024 
00025       ENABLE_VTABLE();
00026 
00027       enum ESensorType {
00028          TYPE_BLACK_WHITE = 0,
00029          TYPE_GRAYSCALE
00030       };
00031 
00032       struct SSensor {
00033          typedef std::vector<SSensor*> TList;
00034 
00035          CVector2 Offset;
00036          ESensorType Type;
00037          SAnchor& Anchor;
00038  
00039          SSensor(const CVector2& c_position,
00040                  ESensorType e_type,
00041                  SAnchor& s_anchor) :
00042             Offset(c_position),
00043             Type(e_type),
00044             Anchor(s_anchor) {}
00045       };
00046 
00047    public:
00048 
00049       CGroundSensorEquippedEntity(CComposableEntity* pc_parent);
00050 
00051       CGroundSensorEquippedEntity(CComposableEntity* pc_parent,
00052                                   const std::string& str_id);
00053 
00054       virtual ~CGroundSensorEquippedEntity();
00055 
00056       virtual void Init(TConfigurationNode& t_tree);
00057 
00058       virtual std::string GetTypeDescription() const {
00059          return "ground_sensors";
00060       }
00061 
00062       virtual void Enable();
00063 
00064       virtual void Disable();
00065 
00066       inline size_t GetNumSensors() const {
00067          return m_tSensors.size();
00068       }
00069 
00070       inline SSensor& GetSensor(size_t un_idx) {
00071          return *m_tSensors[un_idx];
00072       }
00073 
00074       inline SSensor::TList& GetSensors() {
00075          return m_tSensors;
00076       }
00077 
00078       void AddSensor(const CVector2& c_offset,
00079                      ESensorType e_type,
00080                      SAnchor& s_anchor);
00081 
00082       void AddSensorRing(const CVector2& c_center,
00083                          Real f_radius,
00084                          const CRadians& c_start_angle,
00085                          ESensorType e_type,
00086                          UInt32 un_num_sensors,
00087                          SAnchor& s_anchor);
00088 
00089    protected:
00090       
00091       ESensorType ParseType(const std::string& str_type) const;
00092 
00093    protected:
00094 
00096       SSensor::TList m_tSensors;
00097 
00098    };
00099 
00100 }
00101 
00102 #endif
```

---

Generated on 10 Jul 2018 for ARGoS by 
 1.6.1
